# Colorectal Cancer Immunotherapy: Options and Strategies

**DOI:** 10.3389/fimmu.2020.01624

**Published:** 2020-09-18

**Authors:** Nor Adzimah Johdi, Nur Fazilah Sukor

**Affiliations:** UKM Medical Molecular Biology Institute (UMBI), National University of Malaysia, Bangi, Malaysia

**Keywords:** colorectal carcinoma, immunotherapy, FDA, T-cells, antibodies, cytokines, treatments

## Abstract

Colorectal cancer is the third most common cancer in the world with increasing incidence and mortality rates globally. Standard treatments for colorectal cancer have always been surgery, chemotherapy and radiotherapy which may be used in combination to treat patients. However, these treatments have many side effects due to their non-specificity and cytotoxicity toward any cells including normal cells that are growing and dividing. Furthermore, many patients succumb to relapse even after a series of treatments. Thus, it is crucial to have more alternative and effective treatments to treat CRC patients. Immunotherapy is one of the new alternatives in cancer treatment. The strategy is to utilize patients’ own immune systems in combating the cancer cells. Cancer immunotherapy overcomes the issue of specificity which is the major problem in chemotherapy and radiotherapy. The normal cells with no cancer antigens are not affected. The outcomes of some cancer immunotherapy have been astonishing in some cases, but some which rely on the status of patients’ own immune systems are not. Those patients who responded well to cancer immunotherapy have a better prognostic and better quality of life.

## Introduction

Colorectal cancer (CRC) is the third most common cancer in the world with an incidence of 10.2% and a mortality rate of 9.2% of all cancers (1, 2). Approximately 1.8 million new cases and mortality of 0.88 million were recorded until 2018. From 1975 to 2013, CRC incidence rate increased from 10–15 cases per 100,000 population of Americans between the age of 20–49 years old (3). In developing countries such as Argentina, Brazil, and China, the incidence and mortality rate due to CRC increased around 20% (4). Furthermore, the prognosis for patients with metastatic CRC remains poor with a median 5-year survival of only 18.5% in the United States and 27.7% in Europe (1, 2).

Standard conventional treatments for CRC are surgery, chemotherapy and radiotherapy. Depending on the localization and progression of the disease, these treatments can be used in combination (5–8). Total mesorectal excision (TME) through laparoscopic and transanal surgery approaches are often the options for localized cancer and whenever the tumor location is easy to access (9–11). However, complete removal of all cancer cells is often not possible. About 66 and 61% of stage II and III colon and rectal patients underwent further treatments with adjuvant chemotherapy and/or radiotherapy, respectively (12). These treatments have many side effects due to their unspecificity and cytotoxicity toward any cells that are growing and dividing (13, 14). Furthermore, 54% of patients relapse even after neoadjuvant treatment (15). Thus, it is crucial to have more alternative and effective treatments to treat CRC patients.

Cancer immunotherapy is one of the new alternatives in cancer treatment. In comparison to the standard treatment, this treatment manipulates and utilizes patients’ own immune system in combating the cancer cells. The innate and adaptive immune responses are alerted to recognize the cancer cells and potentially eradicate the tumor (16–18). Many successes have been reported especially in hematological malignancies and solid tumors (19, 20). Cancer immunotherapy overcomes the issue of specificity which is the major problem in chemotherapy and radiotherapy. Cancer immunotherapy targets cancer antigens on the malignant cells specifically, alerts the immune systems to the presence of foreign substances and eradicates cancer through the concert of immune responses. The normal cells with no cancer antigens are not affected. The outcomes of some cancer immunotherapies have been astonishing in some cases, but some which rely on the status of patients’ own immune systems are not. Those patients who responded well to cancer immunotherapy have a better prognostic and better quality of life.

Historically, cancer immunotherapy started in 1866 where notable tumor shrinkage was observed among cancer patients who were diagnosed with erysipelas caused by *Streptococcus pyogenes* (21, 22). Later in 1891, William Coley who is known as the ‘Father of Immunotherapy’ continued the discovery journey by introducing heat-inactivated Streptococcal bacteria (Coley’s toxin) into unresectable osteosarcoma patients with the hope that any side effects produced from the infection would shrink the tumor (23). The approach was successful for a time. The patients who developed erysipelas went into spontaneous remission (24, 25). Following this, Coley improved the formulation by combining live and attenuated *Streptococcus erysipelas* and *Bacillus prodigiosus* (26). Around 1000 patients were successfully treated using this method. After 8 years of hard work, Coley’s toxin was commercially available in 1899 (26). However, patients who underwent this treatment were exposed to extremely pathogenic bacteria. Furthermore, due to its unreproducible results, Coley’s toxin was opposed by most of the health practitioners. Surgery remained the most preferable way to treat cancer during that time (27).

After nearly two decades, immunotherapy once again captured scientists’ attention with the new concept of tumor-specific antigens which was found in a mouse model. This was followed by theories on acquired immunological tolerance and immunosurveillance (28–30). A year later in 1957, another cancer immunotherapy approach using interferon-α, a type of cytokine was introduced (31). The first cancer vaccine was also discovered during this era when 25 out of 114 (22%) gynecologic cancer patients went into remission upon treatment with adjuvant tumor lysate (32). In the subsequent years, novel findings on the importance of T cells in cancer immunity made cancer immunotherapy more exciting, thus lead to the discovery of dendritic cells and natural killer cells’ activities in mouse models (33–36). The first monoclonal antibody production using the hybridoma technique was also initiated in 1975 by Koehler and Milstein. They both were awarded a Nobel Prize in 1984 for this crucial finding which is widely used until today (37). Another significant finding in cancer immunotherapy was the discovery of the first immune checkpoint inhibitor namely CTLA-4 in 1988, which led to its first clinical trial in the year 2000 and approval by United States Food and Drugs Administration (FDA) to treat metastatic melanoma in 2011 (38). The emergence of cancer immunotherapy continued until the FDA-approved Interleukin-2 and the first monoclonal antibody (mAbs), Rituximab were used as anti-cancer therapies in 1992 and 1997, respectively (39, 40).

In the 20th century, the FDA has approved various types of immunotherapeutic drugs including Sipuleucel-T, a cancer vaccine to treat castration-resistant prostate cancer in 2010 (41, 42). Five years later, the first oncolytic virotherapy agent known as T-VEC was approved in treating metastatic melanoma (43). The chimeric antigen receptor (CAR) T-cell therapy was also introduced to relapsed B-cell acute lymphoblastic leukemia and diffuse large B-cell lymphoma patients in 2017 and 2018 after getting approval (44, 45). In the same year, Tasuku Honjo and James Allison received their Nobel Prize in Physiology due to their significant contributions in discovering the immune checkpoint inhibitors, PD-1 and CTLA-4, respectively (46). Currently, with an increasing number of FDA approved single and combinational immunotherapeutic drugs over the years, the cancer immunotherapy field is continuously showing potential in treating various types of malignancies.

## Immune Classification

Cancer immunotherapies are classified based on the types of immune mechanisms that are involved either through passive or/and active mechanisms or based on antigen specificity (47). Passive immunotherapies are tumor-targeting mAbs, adoptive cell transfer (ACT) and oncolytic virotherapy while active immunotherapies are immunomodulatory mAbs, anti-cancer vaccines, immunostimulatory cytokines, inhibitor of immunosuppressive metabolism, pattern recognition receptor (PRR) agonists, immunogenic cell death inducer and other non-specific immunotherapeutic agents.

### Monoclonal Antibodies (mAb)

Monoclonal antibodies (mAbs) are immunoglobulin molecules, which are made up of antigen-binding fragments that are connected to a constant region with two identical light and heavy chains. The light chains are made up of one variable and one constant domain while the heavy chains consist of one variable and three constant domains (48). There is also a special region within the variable domain with 3 loops known as the complementarity determining region (CDR) (48).

Initially, the hybridoma technique (49) and phage display (50) were used in producing murine mAbs in the laboratories. With advanced technologies, three types of antibody-engineered mAbs are produced using the same technique, namely chimeric, humanized and human monoclonal antibodies. Chimeric mAbs consist of cloned human amino acids at the constant domain whereas mouse amino acids are located at the variable domain. Chimeric mAbs can be produced by directly joining the variable region immunoglobulin of selected mouse hybridoma into the human constant region through *in vitro* cell-based technology (51). Examples of FDA approved chimeric mAbs are Rituximab that is used to treat Non-Hodgkin Lymphoma, Cetuximab to treat colorectal cancer and Dinutuximab that is used among neuroblastoma patients (52). On the other hand, humanized mAbs are generated through the addition of murine CDR into the human’s variable and constant domain through CDR grafting (53). Some of the FDA approved humanized mAbs are Trastuzumab used to treat breast cancer, Alemtuzumab to treat chronic myeloid leukemia and Bevacizumab to treat colorectal cancer (52). The third type of mAbs is the human mAbs which are produced when the whole mAbs are made up from human amino acids (54). This may be done through the preference of human antibody fragments from human hybridomas and *in vitro* libraries by transgenic mice (48). Human mAbs that are approved by FDA for cancer treatments are Panitumumab to treat colorectal cancer, Ofatumumab to treat chronic lymphocytic leukemia and Ramucirumab to treat gastric cancer (52).

**FIGURE 1:**
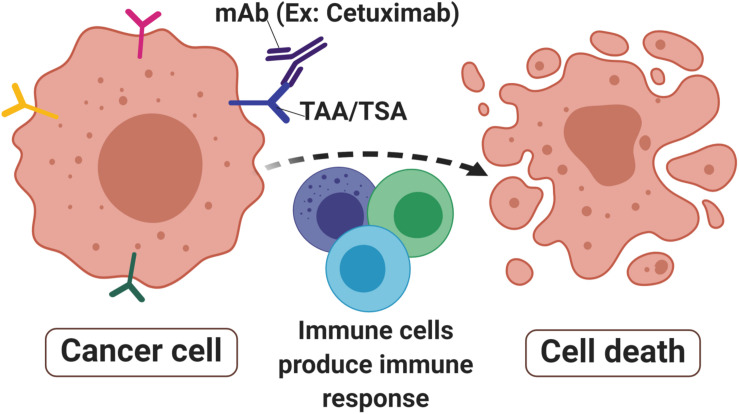
Monoclonal antibodies in cancer. Monoclonal antibodies (mAbs) such as Cetuximab are designed to target tumor-associated antigens (TAAs) or tumor-specific antigens (TSAs) that are abundant in cancer cells surface. The signals produced by receptor activities mediated immune cells toward malignant sites thus produced immune responses that lead to cell death to eradicate the tumor.

Unlike polyclonal antibodies that bind to multiple epitopes, mAbs have a monovalent affinity which makes them bind to an epitope of antigens (55). The mAbs can recognize and bind specifically to tumor antigens of tumor-specific antigen (TSA) (56) or tumor-associated antigen (TAA) (57) that are present on cancer cells surface (Figure 1). TSAs are a group of mutated proteins due to somatic mutations and relatively are restricted to tumor cells (58). Their specificity in tumor cells makes them a good candidate for immunotherapy. One of the good examples of a TSA is the mutated p53 protein that is present in many cancer cells including colorectal cancer. As a result, the p53 synthetic long peptide vaccine was designed to treat metastatic CRC patients (59). The results show that around 90% of the respondents treated with this vaccine produced p53-specific-T cell response with low-grade toxicity suggesting that p53 is indeed one of the attractive TSAs in cancer immunotherapy (59).

In contrast, TAAs have differentially expressed proteins that are present in both malignant and non-malignant cells. Although TAAs are expressed on normal cells, their expression on malignant cells has a unique characteristic resulting in specific immunogenicity (60). Nevertheless, because the antigens are also expressed on the normal cells, they may induce autoimmunity in the host (61). To overcome this, the self-antigen concept suggests that the self-reactive T cells are to be deactivated, therefore increasing their specificity in targeting the unique TAA on tumor cells (62). Furthermore, cancer vaccines must have a high tendency to specifically bind to tumor antigens and effectively kill them with minor adverse effects on normal cells. TAAs can be divided into four categories based on their expression pattern namely cancer-testis antigens (CTA), differentiation antigen, oncofoetal antigen and overexpressed antigens (62). CTA are aberrantly expressed protein in cancer and testis tissues with restricted expression in other normal tissues (63). A widely studied CTA in CRC is the melanoma-associated antigen (MAGE) group, particularly MAGE-A (64), MAGE-A-12 (65), and MAGE-A3 (66) variants. Differentiation antigens are expressed during cell differentiation stages such as Mucin 1 glycoprotein (MUC1) (61) and epithelial cell adhesion molecule (EpCAM) (67). These antigens may not create major side effects as they are tissue-specific and are only expressed during the cell differentiation stage (62). Oncofoetal antigens such as 5T4 (68, 69) and carcinoembryonic antigens (CEA) (70) are found in fetal tissues during its development as well as in several malignancies including ovarian, colorectal and breast cancers. Overexpressed antigens are huge, various groups of common proteins that can be found in both malignant and normal cells. However, they are highly expressed in malignant cells compared to the normal which thus have potential as an immunotherapeutic target (71). Some of the extensively studied overexpressed antigens in CRC are epidermal growth factor receptor (EGFR) (72), coiled-coil domain containing 34 (CCDC34) (73) and RAS-related protein (Rab-1A) (74). The binding of mAbs to TSA or TAA produces molecular signals to immune cells such as T cells, B cells and natural killer cells. This further initiates and activates receptors activities that lead to apoptosis and tumor-killing (75, 76).

The two major types of mAbs are tumor-targeting mAbs and immunomodulatory mAbs.

#### Tumor-Targeting mAbs

One of the passive immunotherapies is tumor-targeting mAbs and it is the most commonly mAbs used in treating hematological malignancies and other solid tumors. There are 76 mAbs that are approved by the European Medical Agency (EMA) and Food Drugs Administration (FDA) for therapeutic use (status 2017) (77). Table 1 listed the top 10 current EMA and/or FDA approved mAbs that are used for cancer treatments.

**TABLE 1:** The top 10 current EMA and/or FDA approved mAbs that are used for cancer treatments (78, 79).

**No.**	**Trade name**	**International Non-proprietary Name (INN)**	**Target**	**Type**	**EMA approval (Year)**	**FDA approval (Year)**	**Cancer**
1	Bavencio^®^	Avelumab	PD-L1	Human IgG1/_K_	Not approved	2017	Metastatic Merkel cell carcinoma
2	Imfinzi^®^	Durvalumab	PD-L1	Human IgG1/_K_	Not approved	2017	Metastatic urothelial carcinoma
3	Lartruvo	Olaratumab	PDGFR-α	Human IgG1	2016	2016	Sarcoma
4	Darzalex^®^	Daratumumab	CD38	Human IgG1/_K_	2016	2015	Multiple myeloma
5	Empliciti	Elotuzumab	SLAMF7	Human IgG1	2016	2015	Multiple myeloma
6	Portrazza	Necitumumab	EGFR	Human IgG1	2016	2015	Non-small cell lung cancer
7	Tecentriq^®^	Atezolizumab	PD-L1	Human IgG1	Not approved	2016	Metastatic non-small cell lung cancer
8	Opdivo	Nivolumab	PD-1	Human IgG4	2015	2015	Non-small cell lung carcinoma; renal cell Hodgkin diseases, melanoma
9	Unituxin	Dinutuximab	GD2	Human IgG1/_K_	2015 (but has been withdrawn)	2015	Neuroblastoma
10	Blincyto^®^	Bevacizumab	CD19	BiTEs	2015	2014	Precursor cell lymphoblastic leukemia-lymphoma

#### Immunomodulatory mAbs

One of the common immunomodulatory mAbs is immune checkpoint inhibitors (ICIs). The ICIs are used to target and/or block immune checkpoints protein ligands on T cells surfaces or other immune cell subpopulations in restoring immune function. The immune checkpoints serve as key regulators that serve as an immune brake when there is sufficient immune response. However, in cancer, there is high activation and overexpression of immune checkpoints leading to suppression of anti-tumor immune response that favors malignant cell proliferation and spread (80, 81).

The most widely studied immune checkpoint targets are programmed cell death 1 (PD-1) and cytotoxic T lymphocyte antigen 4 (CTLA4) due to their overexpression and abundance in various solid tumors and hematological malignancies (82). Nevertheless, other checkpoints are currently being studied for their potential roles in tumor immunity regulation such as lymphocyte activation gene-3 (LAG-3), T cell immunoglobulin-3 (TIM-3), and T cell immunoglobulin and ITIM domain (TIGIT) (83–86).

In colorectal cancer, three FDA-approved ICIs drugs are targeting PD-1 and CTLA-4 (Table 2). However, ICI treatment efficiency is influenced by the microsatellite instability (MSI) status in each CRC patient (87, 88). MSI status is determined through immunohistochemistry staining and polymerase chain reaction targeting 5 MSI markers of BAT25, BAT26, D2S123, D5S346, and D17S250 (89). CRC patients are divided into three groups based on their mutation patterns of microsatellite instability-high (MSI-H), microsatellite instability-low (MSI-L), and microsatellite stable (MSS) (90, 91). ICI drugs targeting PD-1 and CTLA4 are more potent on metastatic CRC patients with MSI-H due to its higher tumor mutation burden (TMB). High TMB is positively correlated with high neo-antigen load thus increasing tumor immunogenicity (92). The majority of CRC patients with MSI-H benefited with this immunotherapy where the disease control rate was 80% using the combination of Nivolumab and Ipilimumab compared to patients having microsatellite stable (93).

**TABLE 2:** FDA approved immune checkpoint inhibitors drugs for colorectal cancer treatments (78).

**Trade name**	**International Non-proprietary Name (INN)**	**Target**	**FDA approval date**	**Cancer**
Keytruda^®^	Pembrolizumab	PD-1	23 May 2017	Unresectable or metastatic mismatch repair deficient (dMMR) and microsatellite instability-high (MSI-H) CRC
Opdivo^®^	Nivolumab	PD-1	1 August 2017	Metastatic mismatch repair deficient (dMMR) and microsatellite instability-high (MSI-H) CRC
Yervoy^®^	Ipilimumab	CTLA-4	10 July 2018	Used in combination with nivolumab Metastatic mismatch repair deficient (dMMR) and microsatellite instability-high (MSI-H) CRC

##### Cytotoxic T-lymphocyte-associated antigen 4 (CTLA-4)

**FIGURE 2:**
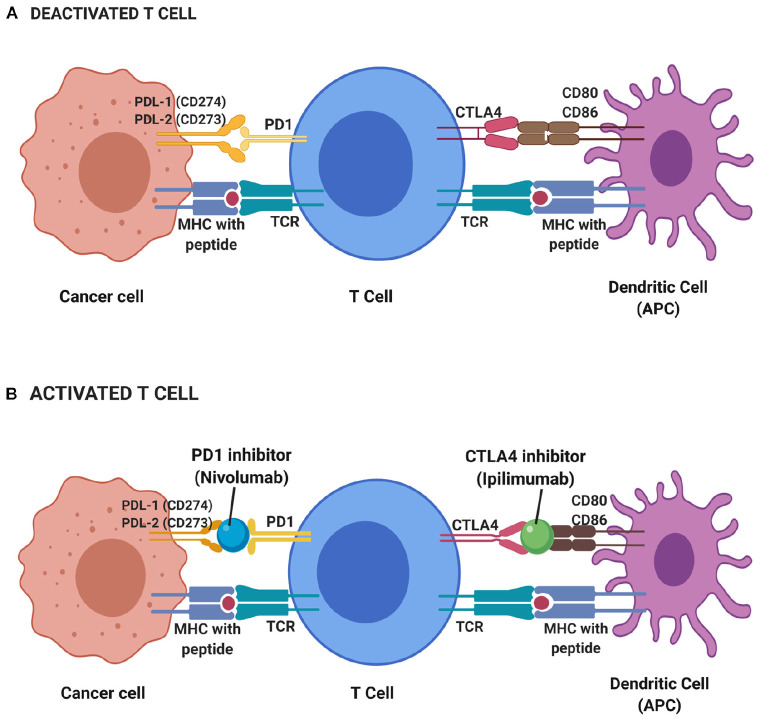
Deactivated **(A)** and activated **(B)** T cell-based on immune checkpoint inhibitors mechanism. During resting of T cell (deactivated T cell), CTLA4 and PD1 receptors on T cell surface binds to CD80 and CD86 on the antigen-presenting cell (APC) such as dendritic cell and PDL-1 and PDL-2 on cancer cell, respectively, while T cell receptor (TCR) binds to major histocompatibility complex (MHC) with the presence of peptide. Thus, no immune response triggered to kill cancer cells. T cells will only be activated with the presence of blockade or inhibitor on CTLA4 and PD1 receptors. Hence, a CTLA4 inhibitor known as Ipilimumab and PD1 inhibitor of Nivolumab functioned to block those receptors and elicit immune response thus leading to the apoptosis of cancer cells.

Cytotoxic T-lymphocyte-associated antigen 4 (CTLA-4) is one of the immune checkpoints that serve as co-inhibitory receptors on the T cell surface (38). The role of CTLA-4 in cancer immunotherapy was discovered through antibody generation that specifically targets the CTLA-4 glycoprotein (94). Under normal circumstances, T cell antigen receptor stimulation is regulated by CD28 co-stimulatory and CTLA-4 co-inhibitory signals (95). When there is an attack from a foreign substance, CD28 co-stimulatory signals increase to stimulate the T cell antigen receptors which further activate the downstream immune signaling (96). When the foreign substance has been cleared, CTLA-4 co-inhibitory signals are activated to stop immune signaling and thus prevent excessive immune responses or autoimmunity. Unlike CD28, CTLA-4 acts as negative regulatory feedback in T-cell stimulation. It switches off the T cell activity during priming and thus inhibits T cell activation and leads to antigenic tolerance. Hence, both CD28 co-stimulatory and CTLA-4 co-inhibitory signals are critical in maintaining T cell homeostasis and self-tolerance (97–99). However, there are some instances where CTLA-4 co-inhibitory signals are constitutively high and this can halt T cell activation (100). Such scenarios are seen in many cases of cancers and suppressor T cells such as T regulatory cells, suggesting the reason for lack of immune responses in cancer patients (101, 102). CTLA-4 protein is homologous to CD28 protein and has a higher binding affinity toward CD80 and CD86 on major histocompatibility complex (MHC) (Figure 2). This has been the avenue in immunotherapy in targeting CTLA-4 protein expression to stimulate the patient’s own T cell immune response. Many immune checkpoint inhibitor drugs are targeting CTLA-4 that is designed to bind and block this protein, thus allow CD28 binding to MHC and stimulate T cell immune response, activate downstream immune signaling and destroy the malignant cells (103, 104).

##### Programmed death 1 receptor and its ligand (PD-1/PD-L1)

Programmed death 1 (PD-1; CD279) is a novel member of the immunoglobulin gene superfamily (IgSF) with restricted expression in the thymus of mice (105). The PD-1 gene was first discovered in apoptosis gene screening and was shown to be involved in apoptosis of interleukin-3 (IL-3)-deprived LyD9 (a murine hematopoietic progenitor cell line) and stimulated 2B4.11 (a murine T-cell hybridoma) (105).

In humans, the PD-1 (PDCD1) gene is located at 2q37.3 which encodes for PD-1 proteins (106). This protein is a type I transmembrane glycoprotein with a size of 50–55 kDa (107). PD-1 is expressed in various immune cells such as CD4 and CD8 T cells, B cells, macrophages, dendritic cells and tumor-infiltrating lymphocytes (TILs) (108, 109). It functions as an immune checkpoint that balances the peripheral tolerance and regulates T-cell responses under normal conditions (110).

Programmed death 1 binds to two ligands, PD-L1 (CD274; B7-H1) and PD-L2 (CD273; B7-DC) with differential expression (111). This binding activates PD-1: PD-L1/L2 pathway which then mediates potent inhibitory signals to hinder the proliferation and function of T effector cells and have inimical effects on antiviral and antitumor immunity (112).

Similar to CTLA-4, the main role of PD-1/PDL1 interaction (PD-1 pathway) under normal conditions is as a brake for immune response in which the pathway can limit T-cell effector responses. One of the ways is through enhancing immunosuppressive regulatory cells (Tregs) development, thus prevent over-activation of the immune response in human peripheral tissues (113). This immune homeostasis is important in protecting us from autoimmune and severe inflammation.

However, this is not always the case in cancer. Overexpression of PD-1 is observed that leads to constant PD-1 (on the T cell surface) binding to its ligand, PDL1 (on the cancer cells). As a result, PD1/PDL1 signals are constitutively high, suppress the activation of T cells and cause antigenic tolerance. This allows immune cell evasion by cancer cells which then support the high tumor proliferation rate (114, 115). Therapeutic strategies targeting PD1/PDL1 pathways have resulted in many checkpoint inhibitors that function to interfere with PD-1/PDL1 binding through competitive binding and lead to restoring effector T cells activity in cancer patients. Drugs inhibitors were designed to bind and block PD1/PDL1 binding to restore effector T cells activation, proliferation, function and downstream immune signaling to destroy the malignant cells (104).

To date, there are five FDA approved PD-1 inhibitor drugs for various cancers (103, 116) (Table 3). The first FDA approved PD-1 inhibitor drug was Pembrolizumab (Keytruda^®^), a humanized monoclonal IgG4 antibody, used in treating melanoma (78). Phase I trial of the drug was tested in solid tumor and hematological malignancies patients. Results show that Pembrolizumab was well tolerated among multiple solid tumors patients (117). There was a significantly longer survival rate with minimum side effects among advanced non-small cell lung cancer (NSCLC) with high PDL1 expression compared to patients treated with platinum-based chemotherapy (118).

**TABLE 3:** FDA approved PD-1 and PDL-1 inhibitor drugs in various types of cancer immunotherapy (78, 116).

**Agent**	**Target**	**Commercial name**	**Date of FDA approval/types of cancer treated**
Pembrolizumab	PD-1	Keytruda^®^	9/2014: Melanoma
			10/2015: Non-small cell lung cancer
			8/2016: Head and neck squamous cell carcinoma
			3/2017: Hodgkin lymphoma
			5/2017: Urothelial carcinoma
			5/2017: MSI-H colorectal cancer
			9/2017: Gastric cancer
Nivolumab	PD-1	Opdivo^®^	12/2014: Melanoma
			11/2015: Renal cell carcinoma
			5/2016: Hodgkin lymphoma
			11/2016: Head and neck squamous cell carcinoma
			2/2017: Urothelial carcinoma
			8/2017: MSI-H colorectal cancer
			9/2017: Hepatocellular carcinoma
Atezolizumab	PDL-1	Tecentriq^®^	5/2016: Urothelial carcinoma
			10/2016: Non-small cell lung cancer
Avelumab	PDL-1	Bavencio^®^	3/2017: Merkel cell carcinoma
			5/2017: Urothelial carcinoma
Durvalumab	PDL-1	Imfinzi^®^	5/2017: Urothelial carcinoma

In CRC patients, Pembrolizumab shows significant benefit to the mismatch-repair deficient or microsatellite instability-high CRC patients (dMMR/MSI-H). Results show the progression-free survival rate up to 78% compared to mismatch-repair proficient, microsatellite stable (pMMR/MSS) patients of 11% (119). Another successful PD-1 inhibitor is Nivolumab (Opdivo^®^) that shows durable responses among the dMMR metastatic CRC (mCRC) cohort of patients. Approximately, 69% of these patients have 12 months of overall survival (OS) (120). Interestingly, a combination of Nivolumab with Ipilimumab (a CTLA4-targeting drug) demonstrates a higher response rate of up to 94% in these patients. This suggests that the combination of immune checkpoints therapy can greatly improve the efficacy of the treatment for dMMR/MSI-H mCRC patients (93).

### Adoptive Cell Transfer (ACT)

Adoptive cell transfer is a cell-based therapy that uses cells either from the patient (autologous transfer) or other donors (allogeneic transfer) to improve immune function (121). There are 3 methods for the ACT; use of tumor-infiltrating lymphocytes (TILs), insertion of chimeric antigen receptor (CAR), and modification of T cell receptors (TCR) (122).

Adoptive cell transfer that uses TILs (ACT-TIL) was first successfully observed in 60% of metastatic melanoma patients who had not been treated with interleukin-2 (IL-2) and 40% of non-respondents IL-2 treatments which resulted in cancer regression (123). However, major drawbacks with ACT-TIL using patients’ own TIL was a limitation in generating tumor-specific T cells. This leads to the development of another approach using patients’ own genetically engineered-TCR. Approximately 13% (2 out of 15) metastatic melanoma patients benefited from this type of ACT with high sustained engineered T cells level were observed after a year of infusion (124). A few years later, ACT uses a better method of CAR insertion. This was shown to be safe to use among a cohort of clear cell renal cell carcinoma (ccRCC) and ovarian cancer patients with no toxicity observed although only two and none of these patients respond toward the treatment, respectively (125, 126).

**FIGURE 3:**
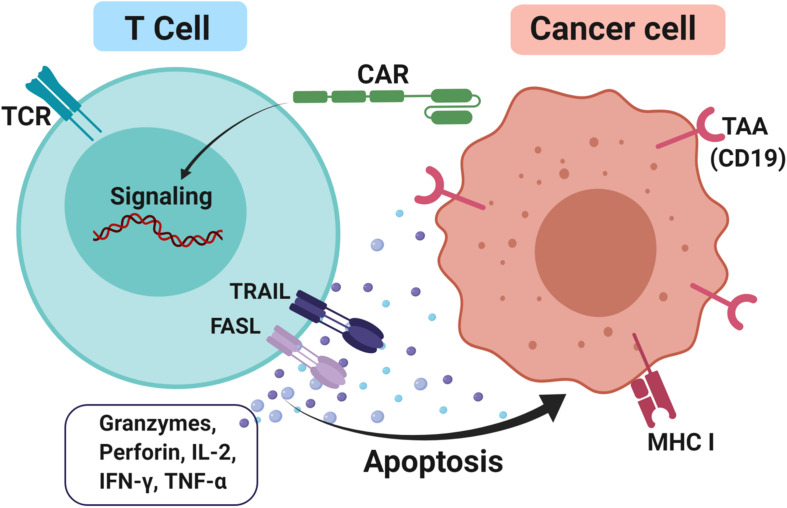
CAR-T cells approach to treating cancer. T cells were firstly isolated either from the patient itself or HLA matched donor through apheresis. Next, the cells were cultured and a genetically modified chimeric antigen receptor (CAR) was inserted into it and these T cells are now known as CAR-T cells. This modification is necessary to enhance T cells’ ability to recognize tumor-associated antigens (TAAs) such as CD19 and avoid the major histocompatibility complex class I (MHC I) restriction recognition on the cancer cell. Upon binding, FAS ligand (FASL) and TNF-related apoptosis-inducing ligand (TRAIL) promotes cytotoxicity by releasing effector cytokines and lead to cancer cell apoptosis.

CAR-T cells consist of antibody variable fragments specific to the antigen of interest that are fused to the isolated patient’s or donor’s T cells (127). In the CAR approach, T cells are isolated from a patient (autologous) or HLA-matched donor (allogeneic), cultured through *ex vivo* and genetically modified through the insertion of the chimeric antigen receptor (CAR) onto the T cells as CAR-T cells (128). The modified *ex vivo* CAR-T cells are re-infused back into the patient and monitored. The modifications are necessary for enhancing the T cells’ ability to recognize the antigen of interest and avoid the major histocompatibility complex restriction recognition. This leads to highly targeted antigen recognition and allows active trafficking to tumor sites, *in vivo* expansion and long-term persistence. Usually, CAR-T cells are engineered toward tumor-associated antigens (TAAs) such as CD19 in diffuse large B cell lymphoma (DLBCL) (129), interleukin 13 receptor alpha-2 (IL13Rα2) (130) and epidermal growth factor variant III (EGFRvIII) in glioblastoma (131) and carcinoembryonic antigen (CEA) in colorectal cancer (132) to promote cytotoxicity and apoptosis (Figure 3). The advantage of CAR-T cells is their specificity in targeting cell surface TAA in an MHC-independent manner. This allows more patients to be treated without the need for MHC-specific treatment. In addition, co-stimulatory domains such as CD28 or 4-1BB (CD137) can be added to improve CAR-T cells’ proliferation and survival rate *in vivo*, thus enhancing the anti-tumor activity of CAR-T cells (133, 134). Furthermore, the T cell responses produce memory cells that help to preserve the immunotherapeutic effect for several years even after treatments (135, 136).

Like many other methods, ACT comes with some limitations. This approach is highly technical and economically challenging for both the industry and patients due to the need to generate tumor-specific lymphocytes for each patient. Secondly, the patient who is receiving the allogeneic transfer is often exposed to the danger of graft-versus-host-disease (GvHD). Furthermore, toxicity is the main issue when targeting antigenic targets such as TAA which are also expressed on normal tissues but are overexpressed on the tumor. An example of these cross-reactivities was seen in CAR-T cells targeting HLA-A^∗^0201–restricted peptide in melanoma-associated antigen (MAGE)-A3. This approach had caused severe damage to gray matter in the brain as the TCR recognized a different but related epitope that was expressed at low levels in the normal brain cells (137). Another limitation is related to cytokine release syndrome (CRS), which is life-threatening toxicity that has been observed in the ACT. CRS refers to an elevated state of circulating level of cytokines, chemokines and other signaling proteins including interleukin 6 (IL-6) and interferon γ as a result of tumor lysis. This, in turn, activates more immune cells and other immune signalings, leading to excessive immune responses and toxicities (138). Other side effects including neurological problems such as problems remembering words, difficulty in speaking, being less alert, hallucinations, seizures and coma. In many patients, these problems fade on their own in a few days, but some have died from these problems. Nevertheless, FDA approved ACT was listed for many hematological malignancies (Table 4) but most of the solid tumors are still undergoing clinical trials.

**TABLE 4:** FDA approved CAR T cells treatments in cancers (78).

**No.**	**Types of cancer**	**CAR T cells**	**Description**	**FDA approval date**	**References**
1	B-cell acute lymphoblastic leukemia (B-ALL) Diffuse large B-cell lymphoma (DLBCL)	Tisagenlecleucel (Kymriah)	CART19 product composed of an extracellular CD19 targeting scFv fused to CD137 and CD3z intracellular signaling domains	30th August 2017	(45, 129, 139, 140)
2	Non-Hodgkin lymphoma	Axicabtagene ciloleucel (Yescarta)	Formerly known as KTE-C19. CART19 product composed of an extracellular CD19 targeting scFv fused to CD28 and CD3z intracellular signaling domains	18th October 2017	(44, 141)

In CRC, CAR-T cells target carcinoembryonic antigens (CEA) and guanylyl cyclase C (GUCY2C) (142, 143) tumor-associated glycoprotein (TAG72) (144), epithelial cell adhesion molecule (EpCAM) (145), NK cell surface receptor ligands (NKG2DLs) such as major histocompatibility complex (MHC) class I-related chain A and B (MICA and MICB, respectively) and six unique long 16 binding protein (UL-BP1-6) (146). The CAR-T cell therapy can work effectively only if these targets are highly expressed in colorectal carcinoma tissues with low expression in other normal tissues. Normally, these molecules are present at low or undetectable levels on normal cells but rapidly appear on the surface of stressed, malignant transformed and infected cells. For example, therapeutic potential targeting CEA was observed with tumor regression and antigen specificity but with some CRS toxicity and severe colitis (143, 147, 148). These effects were due to the overexpression of CEA in both colorectal adenocarcinomas and several normal colonic mucosae of the gastrointestinal tract (149). Despite some of the drawbacks of CEA targeted CAR-T cell approaches, a phase I trial was performed among CEA + mCRC patients to evaluate its efficacy. Interestingly, there were no CAR-T related severe adverse events observed among all the 10 patients involved in this study (150). Furthermore, combination treatment between CEA-CAR-T cells in addition to recombinant human interleukin 12 (rh-IL12) in mouce models show effective and elevated anti-tumor activity of CAR-T cells among various types of solid tumors including CRC (151).

However, some limitations are encountered in effective targeting of solid tumors by CAR-T cells. This explains why CAR-T cells have not proceeded into commercialization and approval for solid tumors. One of the challenges is the efficient trafficking and infiltrating of solid tumors. The microenvironment of solid tumors contains an abundant fibrous matrix and immunosuppressive cells, which protects the tumor tissue and resists immune cell attack. This includes certain chemokines such as CXCL1, CXCL12, and CXCL5 that are secreted by the tumor cells to inhibit the effective delivery of the CAR-T cells (152, 153). Therefore, overcoming this hindrance is through engineering chemokine receptor (CXCL1 receptor) – T cells. This has greatly drive CAR-T cells to migrate toward chemokines secreted-tumor cells (154). However, having the CXCL1 receptor- engineered T cells is not an ultimate solution. Even if the CAR-T cells successfully traffick and infiltrate the cancer cells, the nature of the tumor and environment themselves further inhibit the actions of the CAR-T cell. This refers to the architecture of the cancer cells such as extensive vascular leakage, poor integrity of tissue structure, hypoxia and low pH. In hypoxic conditions, the acidic tumor microenvironment lacks the necessary essential amino acids. Thus, T cells are likely to experience anergy, exhaustion, senescence and stemness, making it a challenge to achieve the desired CAR-T cells tumor killing (155). Also, other immune suppressor cells such as T cells (Tregs), myeloid-derived suppressor cells (MDSCs) and tumor-associated macrophages are present in the tumor microenvironment that further inhibit the CAR-T cells from being activated and producing a response toward cancer cells. Furthermore, immune checkpoint receptors on tumor cells or immunosuppressive cells are able to inhibit T cells by binding to negative regulatory ligands on T cells. As an example, PD-L1 on the tumor cell surface binds to PD1 on T cells, which will inhibit CAR-T cell activation. Lastly, the tumor-derived inhibitory cytokines such as transforming growth factor-β (TGF-β) are able to deter the function of CAR-T cells in killing the cancer cells. TGF-β plays a major role in alleviating the antitumor response where it can downregulate CD8 + effector T cell function and upregulates Treg maturation (156).

Modification of T cell receptors (TCR) is another approach for ACT and it is known as TCR transduced therapy. It is quite similar to CAR-T cells but their mechanisms for recognizing antigens are quite different. In CAR-T cells, antibody fragments are employed to bind the specific antigens on the surface of cancer cells. In contrast, TCRs use heterodimers consisting of alpha and beta-peptide chains to recognize polypeptide fragments presented by MHC molecules (157). This allows recognition of an intracellular, cell surface antigen or a neo-antigen produced by tumor cells after mutation. The TCR-T cell therapy directly modifies TCR binding to tumor antigens with high-affinity by genetic engineering technology (158). Therefore, it requires the identification of specific targets on cancer cells to ensure minimal off-target effects and cross-reactivity in other cells.

In TCR-T cell therapy, the MHC-dependent allows more antigen targeting compared to CARs. These antigens include MART-1, Gp100, CEA, NY-ESO-1, MAGE-A3, MAGE-A4, and others, which are suitable for TCR-T cell therapy (Table 5). In addition, TCR can also be targeted toward neoantigens generated by random mutations in tumor DNA and cancer-testis antigens (159).

**TABLE 5:** Current clinical trials of TCR-T therapy for solid tumors with reported outcomes.

**Target**	**Disease**	**Clinical trial number**	**Phase**	**Response**	**Country**	**References**
MART-1	Metastatic melanoma	NCT00091104	II	30% objective antitumor response	United States	(160)
Gp100	Metastatic melanoma	NCT00923195	II (completed)	19% objective antitumor response	United States	(160)
CEA	Metastatic colorectal	NCT00923806	I (Terminated)	Decreases in serum CEA levels (74–99%) and one patient had an objective regression. Severe transient inflammatory colitis all three patients.	United States	(143)
NY-ESO-1	Metastatic melanoma/synovial cell sarcoma	NCT00670748	I	2 complete remission, 1 partial remission	United States	(161)
NY-ESO-1	Multiple myeloma	NCT01352286	II	80% remission Objective response was 80% at day 42. At year 1, 52% of patients were disease progression-free, 11 were responders. No fatal serious adverse events.	United States	(162)
MAGE-A3	Metastatic melanoma/multiple myeloma	NCT01350401 and NCT01352286	III/IV	2 dies for cardiac toxicity	United States	(163)
MAGE-A4	Esophageal cancer	Registered in the UMIN Clinical Trials Registry as ID: UMIN000002395.	I	7/10 tumor regression	Japan	(164)

Normally, the affinity of human TCRs toward cancer antigens is relatively low, which makes it impossible to recognize and kill tumors effectively. With the advance of engineering technology, genetically-modified TCR is encoded in T cells that give it better specificity and affinity toward cancer antigen. For example in the multiple myeloma patients, TCR was engineered through modifications of several key amino acids in order to have a higher affinity for cancer antigens, NY-ESO-1 (165). The clinical trial showed that 80% of these patients had a good clinical response while 70% of them had a complete or near-complete response (165). Another example is TCR-T cell therapy toward MART-1 for the treatment of metastatic melanoma patients. Results in clinical trials showed higher antitumor reactivity with objective cancer regressions were seen in 30 and 19% of patients who received the human or mouse TCR, respectively. However, patients suffered some acceptable side effects without CRS (160). These findings show that T cells expressing highly reactive TCRs could mediate cancer regression in humans and target rare cognate–antigen-containing cells throughout the body. This is an important implication for the gene therapy of cancer. The reported outcomes of clinical TCR-T cell therapies are listed in Table 5. The first report on TCR-T cell therapy in colon cancer was targeting CEA antigens where some evidence of clinical response was seen but with severe colitis due to the presence of CEA in normal cells in the colon (143). This demonstrates the feasibility of T-cell therapy in metastatic colon cancer, but also the limitations of targeting CEA as an antigen. Following the promising results, another attempt was developed for treating advanced metastatic colon cancer patients. This was using mRNA-engineered T Cells targeting transforming growth factor β- receptor type II (TGFβII) frameshift antigen which is expressed in microsatellite instability positive (MSI+) colon cancer (166). However, the clinical trial was terminated due to severe adverse effects. Other ongoing TCR therapies are against KRAS G12V + tumor (NCT03190941) and KRAS G12D + tumor (NCT03745326), both are at clinical trials phase I/II (166).

T cell receptors therapy although it is a very promising approach comes with many challenges including good targets selection, specific TCRs search, optimal TCR affinity screening, safety evaluation, time and cost (159). In addition, since TCR therapy is highly dependent on MHC for peptide presentation, it may escape immune surveillance due to the downregulation or mutation of MHC molecules in the tumor environment, resulting in clinical limitations. Furthermore, hybridization (mismatch) between exogenous and endogenous chains may occur and induce harmful recognition of self-antigens, leading to graft-versus-host disease (167). Although higher TCR affinity offers a great benefit, there is a possible risk of false targeting. In a nutshell, TCR-T cell therapy has shown some therapeutic potential but there are still many limitations that should be considered carefully.

### Cancer Vaccines

Cancer cells express altered self-antigens that induce weaker responses compared to foreign antigens such as infectious agents. Often, immune stimulants and adjuvant are incorporated together with the cancer vaccines to enhance the effects. Cancer vaccine includes autologous patient-derived immune cell vaccines, tumor antigen-expressing recombinant virus vaccines, peptide vaccines, DNA vaccines and heterologous whole-cell vaccines derived from established human tumor cell lines (168).

Preventive (prophylactic) and therapeutic are the approaches of cancer vaccines treatments in which preventive cancer vaccines intended to minimize cancer incidence, morbidity and mortality while therapeutic vaccines aimed to treat current malignancies and may prevent (169, 170). To date, there are three FDA approved therapeutic cancer vaccines (Table 6).

**TABLE 6:** FDA approved and clinical trial cancer vaccines (78).

**Types of vaccine**	**Trade name, manufacturer**	**Indication/Study details**	**FDA approval date**	**References/Clinical trial identifier**
Attenuated bacteria	BCG Live (TICE, Merck) Previously (TheraCys^®^, Sanofi)	• Treatment and prophylaxis of carcinoma *in situ* (CIS) of the urinary bladder. • Prophylaxis of primary or recurrent state Ta and/or T1 papillary tumors following transurethral resection (TUR).	TheraCys – 21 May 1990. TICE– 16 December 2010	(172, 173, 194)
Autologous patient-derived immune cell vaccine	Sipuleucel-T (PROVENGE, Dendreon)	• Asymptomatic or minimally symptomatic metastatic castrate-resistant (hormone-refractory) prostate cancer (mCRPC)	29 April 2010	(42, 195–198)
Oncolytic virotherapy	Talimogene laherparepvec (IMLYGIC, Amgen Inc.)	• Local treatment of unresectable cutaneous, subcutaneous, and nodal lesions in patients with melanoma recurrent after the initial surgery.	27 October 2015	(43, 199, 200)
Tumor antigen-expressing recombinant virus vaccines	PSA-TRICOM (PROSTVAC-V/F)	• Prostvac is safe and well-tolerated in asymptomatic or minimally symptomatic metastatic castration-resistant prostate cancer	Phase III clinical trial completed	(190, 201–203) NCT01322490
Peptide vaccines	CEA and mammary type mucin (MUC1), (PANVAC-V/F)	• PANVAC-V and PANVAC-F plus sargramostim vaccination among metastatic CRC versus non-CRC, breast and ovarian cohorts	Phase I clinical trial completed	NCT00088413

The first FDA approved cancer vaccine was TheraCys^®^ (Sanofi) (171). It is an intravesical, attenuated Connaught strain of Bacillus Calmette-Guarin (BCG) derived from *Mycobacterium Bovis*. TheraCys^®^ is used in the treatment and prophylaxis of urothelial carcinoma-*in situ* (CIS), particularly non-muscle invasive bladder cancer (NMIBC) subtype. Results showed that approximately 74% of the patients showed a complete response with BCG compared to Doxorubicin (172). This vaccine also improves the protection against superficial bladder cancer recurrence. A randomized trial demonstrated that 70% of the CIS patients have a complete response to BCG therapy instead of doxorubicin suggesting the effectiveness of this vaccine compare to chemotherapy (173). At the start, two BCG strains were available; Connaught (TheraCys^®^, Sanofi) and Tice (TICE^®^, Merck). However, due to the supply shortage of TheraCys^®^, it was discontinued and replaced by TICE^®^. Nevertheless, a study conducted previously shows that treatments using different BCG strains among NMIBC patients have a significant role as BCG Connaught treatment was more effective than BCG Tice in terms of 5 years recurrence-free survival rate (174). Furthermore, a cohort study among 2099 NMIBC patients using both strains indicates that Connaught was more effective than Tice among patients without BCG maintenance (175).

Another FDA approved therapeutic vaccine is Sipuleucel-T (Provenge; Dendreon) which used to treat prostate cancer patients who are asymptomatic or minimally symptomatic metastatic castration-resistant. This vaccine uses the patient’s own immune cells (dendritic cells, T cells, B cells and natural killer cells) isolated through leukapheresis. The immune cells were cultured and incubated with PA2024, a fusion protein made up of prostatic acid phosphatase (PAP) which is linked to granulocyte-macrophage colony-stimulating factor (GM-CSF) as the adjuvant (41, 176). Antigen-MHC complex was presented on activated DCs surface during incubation thus inducing CD4+ and CD8+ cells to act against PAP while GM-CSF improves DCs maturation upon being introduced into patients (177). This vaccine also shows significant survival benefit (50% higher than in control) for this population of asymptomatic patients who have not been treated with chemotherapy, except for docetaxel (whose inherent toxicities often lead patients and physicians to delay administration until symptoms develop) (42). Currently approaches to enhance efficacy are considered to increase the efficiency of Sipuleucel-T in a wider range of patients. A study to look at the adverse effects related to the usage of this vaccine was performed and an adverse event spectrum was consistent with no new safety concerns observed from 2010 to 2017 (178).

Talimogene laherparepvec (T-VEC) (IMLYGIC^®^, Amgen Inc.) is the latest FDA approved vaccine to treat unresectable cutaneous, subcutaneous, and nodal lesions melanoma recurrent before initial surgery (179). T-VEC is a genetically modified oncolytic virotherapy made up of herpes simplex virus type I (HSV-1) (180). Significantly higher (16.3%) durable response rate (DRR) was demonstrated among unresected stage IIIB to IV melanoma patients injected with T-VEC compared to GM-CSF (2.1%) (43). A randomized trial among advance and unresectable melanoma patients revealed that combination therapy of T-VEC and ipilimumab have a significantly higher (39%) objective response rate compared to ipilimumab only (18%) due to their substantial activity (181, 182).

#### CRC Cancer Vaccines

To date, there are no CRC cancer vaccines approved by the FDA. Most of them are still undergoing clinical trials. Many studies are looking at various attractive, overexpressed single and combination uses of TAAs such as CEA (183–185), MAGE (186, 187), and MUC1 (188, 189). One of the cancer vaccine examples is the Poxviral vaccine regimen which targets TAAs such as CEA and MUC-1 using TRICOM vaccination strategy. In this approach, poxviruses are used as a viral vector for targeting CEA and MUC-1 in combination with T-cell co-stimulatory molecules (B7-1, ICAM-1, and LFA-3). The addition of the co-stimulatory molecules has been shown to greatly enhance the immune response to the antigen (170, 184, 190). The recombinant poxviruses were engineered to express both signal 1 (antigen) and signal 2 (co-stimulatory molecules), with each transgene being driven by a different poxvirus promoter. Preclinical studies have demonstrated that when a TAA transgene is placed into a poxvirus genome, its expression leads to a more vigorous host T-cell immune response to the TAA than would be achieved otherwise (191). Approximately 56% of the metastatic carcinoma patients showed a significant immune response toward MUC-1 and/or CEA following this vaccination (184, 190). In a phase I clinical trial of PANVAC-V, PANVAC-F and sargramostim (GM-CSF immunostimulator), this type of vaccine is associated with enhancing immune responses and has shown evidence of clinical activity among advanced CRC versus non-CRC, breast and ovarian carcinoma cohort.

Another peptide-based cancer vaccine targets CEA only. The study which was conducted using transgenic mice has shown that immunization with CEA peptides can elicit an immune response to kill cancer cells and hence may improve survival (192). Vaccination with an anti-CEA can break immune tolerance to TAA CEA and induce anti-CEA antibodies as well as CEA-specific CD4^+^ T-helper responses in colon cancer patients and mice transgenic for human CEA (193). Furthermore, combination strategies with the CTL peptides of CEA to get both CDT-helper and CTL responses in the transgenic CEA/HLA-A2 mouse model have shown overall significant immune responses and survival (192). The combined vaccination strategy results in the potential use of this vaccination strategy for future clinical applications.

### Cytokines

Cytokines are a group of small cell-signaling polypeptides with a molecular weight of less than 30 kDa (204). They are secreted by various cells mainly the immune cells (T cells, neutrophils and macrophages), endothelial cells, fibroblasts, and other stromal cells (205). There are more than 130 cytokines with various roles. However, their main function is similar which is to stimulate and modulate robust immune responses toward inflammations and infections (206). Besides, these glycoproteins can act on the cells that produce them (autocrine action), in adjacent cells (paracrine action) or in distant cells (endocrine action) (207).

Cytokines are one of the potential polypeptides used in immunotherapy since they can enhance patients’ immune responses well. Three recombinant cytokines such as recombinant interferon-alpha and interleukin-2 drugs have been approved by the FDA and EMA for the treatment of several malignancies (Table 7) (208). A renewed interest in the anti-tumor properties of cytokines has led to an exponential increase in the number of clinical trials that explore the safety and efficacy of cytokine-based drugs, not only as single agents but also in combination with other immunomodulatory drugs. These second-generation drugs under clinical development include known molecules with novel mechanisms of action, new targets, and fusion proteins that increase the half-life and target cytokine activity to the tumor microenvironment or the desired effector immune cells. In addition, the detrimental activity of immunosuppressive cytokines can be blocked by antagonistic antibodies, small molecules, cytokine traps or siRNAs.

**TABLE 7:** FDA and EMA approved cytokines drugs for various cancer treatments.

**Cytokines**	**Trade name, manufacturer**	**FDA approval date**	**Type of cancer treated**	**References**
Recombinant interleukin (IL)-2	Aldesleukin, (Proleukin, Chiron)	1992	1. Metastatic melanoma	(209, 210)
			2. Renal cell carcinoma (RCC)	
Recombinant alpha 2a	IFN-α2a (Roferon^®^-A, Roche)	1986	1. Hairy cell lymphoma	(210–212)
			2. Chronic myelogenous leukemia (CML)	
			3. Melanoma (not successful due to toxicity)	
Recombinant alpha 2b	IFN-α2b (Intron^®^ –A, Merck)	1986	1. AIDS-related Kaposi’s sarcoma	(213–216)
			2. Melanoma	
			3. Follicular lymphoma	
			4. Multiple myeloma	
			5. Hairy cell leukemia	
			6. Cervical intraepithelial neoplasm	

## Summary

In this review, we provide an overview of the novel trends in the immunotherapy field that are yielding therapeutic benefits in CRC patients. We summarize the advantages and drawbacks of each type of immunotherapy in Table 8. The future for immunotherapy is vast with different approaches. What it takes is smart strategies in overcoming the evasion of cancer cells from immune recognition.

**TABLE 8:** The type of novel trends in the immunotherapy with their advantages and drawbacks.

**No.**	**Types of immunotherapy**	**Advantages**	**Drawbacks**
1	Monoclonal antibodies (mAbs)	• Relatively cost-effective among all of the other types, therefore it is highly reproducible.	• Labor intensive in order to determine the potential immunotherapeutic targets.
		• Commercializable	• Short half-life mAbs may be less effective after some time
		• High specificity toward targeted antigens	• Some cells produce high protein level and these cells may escape from T cells and survive in the host
		• Effective in treating various types of cancers, regardless of hematological malignancies or solid tumors	
2	Immune checkpoint inhibitors (ICIs)	• Relatively sensitive. Therefore, only minimum doses required for each patient	• The adverse effect such as systemic toxicity is most likely to occur among patients
		• High specificity toward targeted inhibitors	• Not all of the patients may respond toward ICI as some of their T cells are unable to identify and kill malignant cells
		• May enhance patient’s T cell function through the activation mechanism prior blockade	
		• Works best with combination treatment which may increase its efficacy	
3	Vaccine	• Vaccine goes direct to the tumor upon introduced (localized)	• Potential of rejection due to the introduction of foreign materials
4	Oncolytic virus	• The virus only replicates in malignant cells thus lead to apoptosis whereas no virus will be survived in normal cells as they perform virus killing mechanism	• Efficacy may be reduced due to anti-viral immunity
5	Adoptive cell transfer (Chimeric antigen receptor CAR T cells)	• Personalized toward each patient	• Expensive procedures
		• Works better among hematological malignancies patients as CAR T cells may prolong the remission among these group of patients	• Very technical, require highly skilled staff
		• Have immune memory features due to permanent modification done toward the T cells.	• Prone to cytotoxicity toward hosts such as GvHD, CRS and B-cell aplasia
6	Cytokines	• Earliest approaches in immunotherapy and FDA approved drugs	• Prone to cytotoxicity (cytokine storm) among patients due to excess cytokine level
		• It is small in size makes it easier to interfere and disturb cancer cells division	
		• Helps in boosting patients’ immune system function thus promotes T cells to kill the malignant cells effectively	

## Author Contributions

NJ and NS contributed to the conception and design of the study. NS wrote the first draft of the manuscript, looked for the raw material and summarize the findings, and constructed the diagrams and tables. NJ did the final editing and final input of ideas. Both authors contributed to manuscript revision, read and approved the submitted version.

## Conflict of Interest

The authors declare that the research was conducted in the absence of any commercial or financial relationships that could be construed as a potential conflict of interest.
